# Resilience as a Mediator Between Childhood Trauma and Adult Psychopathology: The Moderating Role of Harm Avoidance in Korean Adults

**DOI:** 10.3390/brainsci15121308

**Published:** 2025-12-04

**Authors:** Eun Soo Kim, Young Chul Shin, Yun Tae Kim, Kang-Seob Oh, Sang-Won Jeon, Dong-Won Shin, Junhyung Kim

**Affiliations:** 1 Department of Psychiatry, Kangbuk Samsung Hospital, Sungkyunkwan University School of Medicine, Seoul 03181, Republic of Korea; eunsoo421.kim@samsung.com (E.S.K.); yc523.shin@samsung.com (Y.C.S.); kangseob.oh@samsung.com (K.-S.O.); swj.jeon@samsung.com (S.-W.J.); dwon.shin@samsung.com (D.-W.S.); 2 Workplace Mental Health Institute, Kangbuk Samsung Hospital, Sungkyunkwan University School of Medicine, Seoul 03181, Republic of Korea; 3 Division of Biostatistics, Kangbuk Samsung Hospital, Sungkyunkwan University School of Medicine, Seoul 03181, Republic of Korea; yt9771.kim@samsung.com

**Keywords:** childhood trauma, neuropsychiatric mechanism, resilience, harm avoidance, temperament, depression, anxiety

## Abstract

**Background/Objectives:** Childhood trauma is a well-established risk factor for adult psychopathology, yet the underlying neuropsychiatric mechanisms remain unclear. Therefore, this study examined whether resilience mediates the relationship between childhood trauma and depressive and anxiety symptoms, and whether this pathway is moderated by harm avoidance (HA). **Methods:** A total of 218 Korean adults (aged 19–50 years; 79 men and 139 women) completed validated measures of childhood trauma (Childhood Trauma Questionnaire (CTQ)-Short Form), harm avoidance (Temperament and Character Inventory–Harm Avoidance subscale), resilience (Brief Resilience Scale), depression (Patient Health Questionnaire-9), and anxiety (Generalized Anxiety Disorder-7). Mediation and moderated mediation models were tested using structural equation modeling, and indirect effects were estimated via bootstrapping with 5000 resamples. **Results:** Childhood trauma was associated with lower resilience, an effect moderated by HA. Resilience was strongly inversely associated with depression and anxiety. Indirect effects of trauma through resilience were significant for both outcomes, with stronger effects at higher HA. **Conclusions:** The associations between childhood trauma and both depression and anxiety were mediated by resilience, and this indirect pathway was amplified by HA. These findings suggest a neuropsychiatric mechanism whereby early-life stress and temperament jointly shape effective neural vulnerability, leading to depression- and anxiety-associated outcomes.

## 1. Introduction

Childhood trauma is highly prevalent worldwide, with approximately 17% of children exposed to domestic violence. This results in profound and long-lasting consequences for individuals and society [1,2]. Childhood trauma is consistently associated with early-onset psychopathology and remains a major risk factor for adult mental disorders, including suicide attempts (41%), self-harm (39%), depression (21%), and substance abuse [3,4,5]. Importantly, childhood trauma constitutes a general risk factor as well as one with profound clinical implications, being linked to more severe symptomatology [6], therapeutic resistance [7], and poor prognosis [8]. Emerging neuropsychiatric evidence indicates that early-life trauma exerts long-term effects on brain networks involved in emotion regulation, reward processing, and stress reactivity. This occurs specifically within the hypothalamic–pituitary–adrenal axis and limbic–prefrontal circuits [9,10]. Recent meta-analytic and neuroimaging research has further delineated the pathways through which childhood trauma contributes to adult psychopathology, highlighting increased threat sensitivity [11], impaired emotion regulation [12], and strengthened negative cognitive biases [13]. Despite these well-established associations, important gaps remain in terms of understanding the psychological factors that determine why some trauma survivors develop psychopathology, whereas others maintain psychological health [14]. Therefore, clarifying these neuropsychiatric mechanisms is essential for developing effective preventive and therapeutic strategies for trauma-exposed populations.

Resilience is hypothesized to be a key factor mediating the association between childhood trauma and adult psychopathology. Childhood maltreatment undermines multiple domains of adult resilience, including coping, self-esteem, and emotion regulation [15]. From a neuropsychiatric perspective, resilience reflects the psychological adaptation, neural flexibility, and top-down regulation within corticolimbic and autonomic networks that modulate stress responses [16]. Accumulating evidence suggests that resilience buffers against adverse mental health outcomes. For example, resilience has been shown to mediate and moderate the associations between adverse childhood experiences and post-traumatic stress disorder and depression [17]. In a study of adults with histories of childhood abuse, higher resilience scores predicted reduced hazardous alcohol use (β = −0.11, *p* = 0.0014) and lower illicit drug use (β = −0.03, *p* = 0.0008) [18]. Prior research indicates that resilience mediates the effect of post-traumatic stress symptoms, with this pathway further moderated by the severity of childhood trauma [19]. In the Korean context, validated measures, such as the Korean version of the Brief Resilience Scale (BRS-K), have facilitated reliable assessment [20], and population-based studies have demonstrated robust negative associations between childhood trauma and resilience [21,22,23]. Nevertheless, research examining the factors that influence how trauma affects resilience remains scarce. According to Garmezy and Rutter, resilience is not a fixed personality trait but rather a dynamic construct shaped by developmental, environmental, and psychosocial factors that can be strengthened through interventions [24,25,26]. Because resilience may vary across cultural contexts [26], examining these processes in Korea is particularly informative. Therefore, identifying the risk factors that modulate the trauma–resilience relationship represents an important area of inquiry.

The harm avoidance (HA) temperament trait reflects a heritable tendency toward behavioral inhibition characterized by excessive caution, apprehensiveness, and pessimism. Among the four temperament dimensions proposed in Cloninger’s personality model (HA, Novelty Seeking, Reward Dependence, Persistence), HA, unlike the other subscales with modest links [27,28], shows the most direct associations to serotonergic activity and hyperreactivity of limbic circuits involved in threat sensitivity [29,30], thereby offering a key clue to understanding the mechanisms through which early-life trauma may weaken resilience. However, research on the interaction between childhood trauma and HA remains limited. Longitudinal prospective data from the Northern Finland Birth Cohort 1966 indicate that higher harm avoidance predicts the later onset of major depressive disorder, with significant effects observed in both men (OR = 1.05, 95% CI [1.01–1.09]) and women (OR = 1.09–1.13) over a 23-year follow-up period [31]. Furthermore, cross-sectional studies have also shown an inverse relationship between HA and resilience in adults, with Australian (r = −0.56) and Korean community samples (r ≈ −0.53) both reporting robust effects [22,23,27]. One hypothesis is that HA promotes avoidance of painful internal experiences, thereby hindering the emotional processing and integration of trauma [32,33]. Furthermore, by sustaining anxiety symptoms and reinforcing avoidance behaviors through threat-oriented cognitive biases, HA may further weaken resilience [34]. Collectively, these findings suggest a mechanism in which higher HA fosters experiential avoidance and threat-biased attention, thereby restricting mastery experiences and ultimately eroding resilience. The Diathesis–Stress Model posits that psychopathology arises from the interaction between inherent vulnerability (diathesis) and environmental stressors [35]. Accordingly, HA may represent a constitutional vulnerability that amplifies the detrimental effects of childhood trauma on resilience.

Given that HA may amplify the negative effects of childhood trauma on resilience, this study tested an integrated moderated mediation model. Specifically, the study hypothesized that (1) resilience would mediate the relationship between childhood trauma and psychopathology, and (2) the negative effect of trauma on resilience would be strongest among individuals with high HA, thereby amplifying the indirect pathway to depression and anxiety. In light of the debate concerning the role of HA in responses to trauma exposure, this study offers timely evidence linking temperament and resilience. This study aimed to elucidate an integrative neuropsychiatric model in which early-life trauma, temperament-based vulnerability, and resilience shape mood and anxiety symptoms in relatively healthy adults prior to the onset of psychopathology. Given the substantial global mental health burden attributable to trauma, clarifying this mechanism may inform the development of targeted interventions that enhance resilience among trauma-exposed populations, particularly those characterized by high HA to prevent later psychopathology. Thus, this study examines whether resilience mediates the association between childhood trauma and adult psychopathology in the general population, and whether this indirect pathway is moderated by harm avoidance.

## 2. Materials and Methods

### 2.1. Participants and Procedure

#### 2.1.1. Recruitment, Eligibility, and Data Collection

Participants were 218 adults aged 19–50 years (79 men, 36.2%; 139 women, 63.8%) who were recruited through advertisements posted at Korea University Guro Hospital between 17 August 2023, and 30 January 2024. Recruitment advertisements were distributed in public areas of the hospital (e.g., outpatient lobby, bulletin boards), enabling participation from community-dwelling adults, including hospital visitors and local residents. Upon initial contact, recruitment procedures and informed consent were conducted either in person at the outpatient clinic of the hospital or via telephone contact, followed by electronic consent through a secure e-contract service. Eligibility required that participants (1) be adults aged 19 to 50 years and (2) demonstrate adequate understanding of the study purpose and procedures as well as written informed consent. Exclusion criteria included (1) inability to read the consent form and (2) lack of access to a smartphone or substantial difficulty operating one. After providing written informed consent, participants completed questionnaires, assessing sociodemographic variables (e.g., age, sex, educational level, occupational status) and psychological factors (e.g., childhood trauma history, personality traits, resilience, anxiety, and depression). Ethical approval was obtained from the Institutional Review Board of Korea University Guro Hospital (Institutional Review Board No. K2023-1381-001). All research procedures adhered to the Declaration of Helsinki. The current participants overlapped with participants from an earlier validation of the Korean version of the Adult Stress and Adversity Inventory [36].

#### 2.1.2. Participant Characteristics

Descriptive statistics of the demographic and clinical characteristics of the participants are summarized in Table 1. The mean age of the participants was 29.5 years (SD = 6.02), approximately two-thirds were women (63.8%), and most participants were employed (78.4%). In terms of psychological measures, the mean CTQ score was 39.4 (SD = 14.3), with emotional neglect emerging as the most prominent subtype. The mean HA score was 42.5 (SD = 15.6), and the mean BRS score was 19.8 (SD = 5.4). The mean PHQ-9 and GAD-7 scores were 5.0 (SD = 5.5) and 4.4 (SD = 4.5), respectively, indicating mild symptoms on average but with notable variability. Across the TCI temperament scales, numerically similar mean scores were observed, which likely reflects their comparable item structures and scoring distributions (Supplementary Table S1).

### 2.2. Measures

#### 2.2.1. Childhood Trauma

Childhood trauma was assessed using the Korean adaptation of the Childhood Trauma Questionnaire (CTQ)-Short Form [37]. This 28-item instrument measures five categories of maltreatment: emotional abuse, physical abuse, sexual abuse, emotional neglect, and physical neglect. For the current analysis, only the total score was used; however, the CTQ provides five subscales. Each item is rated on a five-point Likert scale (1 = never true to 5 = very often true), with higher scores indicating more severe trauma exposure. Prior validation research demonstrated acceptable reliability (Cronbach’s α = 0.79) [37], and the internal consistency of the current study sample was excellent (Cronbach’s α = 0.93).

#### 2.2.2. Harm Avoidance

Temperament was measured using the Korean version of the Temperament and Character Inventory-Revised Short (TCI-RS), a 140-item self-report measure rated on a 5-point Likert scale ranging from not at all (0) to very true (4) [38]. The instrument comprises four domains: Novelty Seeking, HAs, Reward Dependence, and Persistence. HA reflects a heritable inclination toward behavioral inhibition characterized by excessive cautiousness, anticipatory worry, and pessimism; higher scores indicate greater HA. In the Korean validation study of the TCI-RS involving 2021 adults, mean HA scores were 35.18 (SD = 10.35), providing an appropriate normative reference for interpreting HA levels in the present sample. The Korean HA scale is reliable (Cronbach’s α = 0.86) [38], and the internal consistency of the current study sample was acceptable (Cronbach’s α = 0.72). Other subscale-related information is presented in Supplementary Table S1.

#### 2.2.3. Resilience

Resilience was measured using the BRS-K [20], which is a six-item measure assessing the capacity to recover from adversity. Responses are rated on a five-point Likert scale (1 = strongly disagree to 5 = strongly agree). In this study, the summed score (range: 6–30) was used, with higher values reflecting greater resilience. Internal consistency was excellent (Cronbach’s α = 0.94) and closely matched that of previous Korean samples (Cronbach’s α = 0.91) [20].

#### 2.2.4. Depressive Symptoms

Depressive symptoms were assessed using the Korean version of the Patient Health Questionnaire-9 (PHQ-9) [39]. This instrument measures the frequency of nine depressive symptoms over the past two weeks, rated on a four-point Likert scale (0 = not at all to 3 = nearly every day). Higher total scores indicate greater depressive symptomatology. Internal consistency was excellent (Cronbach’s α = 0.91) and comparable with that of previous reports (Cronbach’s α = 0.88) [39].

#### 2.2.5. Anxiety Symptoms

Anxiety was assessed using the Korean adaptation of the Generalized Anxiety Disorder-7 (GAD-7) [40]. This seven-item measure evaluates generalized anxiety symptoms over the past two weeks, with items rated from 0 (not at all) to 3 (nearly every day). Higher scores indicate greater anxiety severity. Internal consistency in this study was strong (Cronbach’s α = 0.90) and consistent with that of prior findings (Cronbach’s α = 0.93) [40].

### 2.3. Statistical Analysis

Before model estimation, variable-wise missingness and basic patterns were examined. The overall proportion of missing values was low (<5%). Full information maximum likelihood under a missing-at-random assumption was applied to both endogenous and exogenous variables (fixed.x = FALSE). Scale totals were computed following instrument scoring conventions and held constant across analyses.

With approximately 15 freely estimated parameters, our sample size of 218 yields a subject-to-parameter ratio of roughly 14:1 [41]. This exceeds commonly recommended SEM guidelines (e.g., 5–10 cases per parameter and *N* ≥ 200 for path models) [41]. Therefore, the sample was considered modest but adequate for the hypothesized mediation and moderated mediation analyses.

Structural equation modeling (SEM) using the lavaan package 0.6–16 was employed to test the hypothesized mediation and moderated mediation models. All focal constructs in the path models were specified as observed total scores (CTQ, HA, BRS, PHQ-9, and GAD-7). HA was selected a priori due to its strongest theoretical support for risk-avoidance and stress vulnerability [29,30,31], whereas other TCI subscales were excluded for limited relevance and to avoid unnecessary model complexity. Given their Likert-type formats, total scores were treated as approximately continuous, and models were estimated using robust maximum likelihood with bias-corrected bootstrap standard errors (5000 resamples). Predictors were mean-centered, and the interaction term (CTQ × HA) was formed from centered variables. CTQ and HA were allowed to covary in all models.

In terms of measurement validity, prior validation studies of the CTQ, BRS, PHQ-9, GAD-7, and HA instruments justified the use of total scores in the current path models. Formal item-level confirmatory factor analyses were not included owing to resource constraints; therefore, measurement error was not modeled, which may have attenuated structural path estimates.

**Figure 1:**
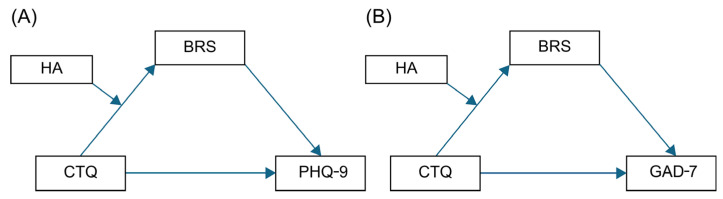
Conceptual frameworks of the moderated mediation effect of Harm Avoidance (HA). Effects on the relationships between Childhood Trauma (CTQ), Resilience (BRS), and (**A**) Depression (PHQ-9) and (**B**) Anxiety (GAD-7). Abbreviations: CTQ, Childhood Trauma Questionnaire; BRS, Brief Resilience Scale; PHQ-9, Patient Health Questionnaire-9; GAD-7, Generalized Anxiety Disorder-7.

The conceptual and analytical models are depicted in Figure 1. In the mediation model, childhood trauma (CTQ) was specified as the independent variable, resilience (BRS) as the mediator, and depressive (PHQ-9) or anxiety (GAD-7) symptoms as dependent variables. In the moderated mediation model, harm avoidance (HA) was modeled as a moderator of the path from CTQ to BRS.

Model adequacy was evaluated using χ^2^, χ^2^/df, comparative fit index (CFI), Tucker–Lewis index (TLI), root mean square error of approximation (RMSEA) with 90% confidence intervals, and standardized root mean square residual (SRMR). Given the known sensitivity of χ^2^ to sample size, interpretation prioritized CFI, TLI, and RMSEA, with findings near conventional thresholds interpreted cautiously and theoretical coherence emphasized over marginal fit improvements.

Indirect effects were estimated using bias-corrected bootstrapping (5000 resamples). For moderated mediation, conditional indirect effects of CTQ on each outcome via BRS were computed at HA = mean ± 1 standard deviation (SD) within the SEM framework. The index of moderated mediation was defined as (b_3_ × c_2_), where b_3_ represents the CTQ × HA effect on BRS and c_2_ represents the BRS effect on the outcome. Statistical significance was inferred when 95% bootstrap confidence intervals excluded zero.

## 3. Results

### 3.1. Correlations Among Main Variables

Bivariate correlations among the main study variables are presented in Table 2. CTQ scores were positively correlated with HA (r = 0.25, *p* < 0.001), PHQ-9 (r = 0.30, *p* < 0.001), and GAD-7 (r = 0.28, *p* < 0.001), and negatively correlated with BRS (r = −0.22, *p* < 0.01). HA showed negative correlations with BRS (r = −0.45, *p* < 0.001) and positive correlations with PHQ-9 (r = 0.42, *p* < 0.001) and GAD-7 (r = 0.40, *p* < 0.001). BRS was inversely correlated with both PHQ-9 (r = −0.48, *p* < 0.001) and GAD-7 (r = −0.46, *p* < 0.001). These results indicate that greater trauma exposure and higher HA are linked to more severe depressive and anxiety symptoms, whereas resilience appears to play a protective role.

### 3.2. Mediation and Moderated Mediation Analyses

#### 3.2.1. Model Fit

Overall, the SEM models demonstrated adequate but mixed fit indices. The depression model (PHQ-9) had the following values: χ^2^ (9) = 31.48, CFI = 0.92, TLI = 0.81, RMSEA = 0.11, and SRMR = 0.06. The anxiety model (GAD-7) had the following values: χ^2^ (9) = 30.13, CFI = 0.93, TLI = 0.83, RMSEA = 0.10, and SRMR = 0.06.

#### 3.2.2. Depression Model (PHQ-9)

Results of the mediation and moderated mediation analyses for depression are summarized in Table 3. CTQ was significantly associated with lower resilience (BRS; β = −0.147, *p* = 0.011), and HA further strengthened this negative association (CTQ × HA: β = −0.161, *p* < 0.001). In turn, resilience was inversely associated with depressive symptoms (β = −0.830, *p* < 0.001). The indirect effect of CTQ on PHQ-9 through BRS was significant (β = 0.122, 95% confidence interval (CI) [0.015, 0.226]), whereas the direct effect of CTQ on PHQ-9 was nonsignificant (β = 0.068, *p* = 0.395). The total effect of CTQ on PHQ-9 remained significant (β = 0.190, *p* = 0.002). These results indicate full mediation, which suggests that resilience transmits the effect of childhood trauma on depression, with HA moderating the CTQ → BRS pathway.

#### 3.2.3. Anxiety Model (GAD-7)

The anxiety outcome results are presented in Table 4. CTQ was associated with lower resilience (β = −0.156, *p* = 0.007), and this effect was also moderated by HA (CTQ × HA: β = −0.115, *p* = 0.008). Resilience was strongly and inversely associated with anxiety symptoms (β = −1.027, *p* < 0.001). The indirect effect of CTQ on GAD-7 through BRS was significant (β = 0.160, 95% CI [0.029, 0.289]), whereas the direct effect was nonsignificant (β = −0.091, *p* = 0.260). The total effect of CTQ on GAD-7 was also nonsignificant. This pattern supports full mediation, with resilience fully transmitting the effect of childhood trauma on anxiety and HA moderating the CTQ → BRS link.

### 3.3. Conditional Effects of HA

**Figure 2:**
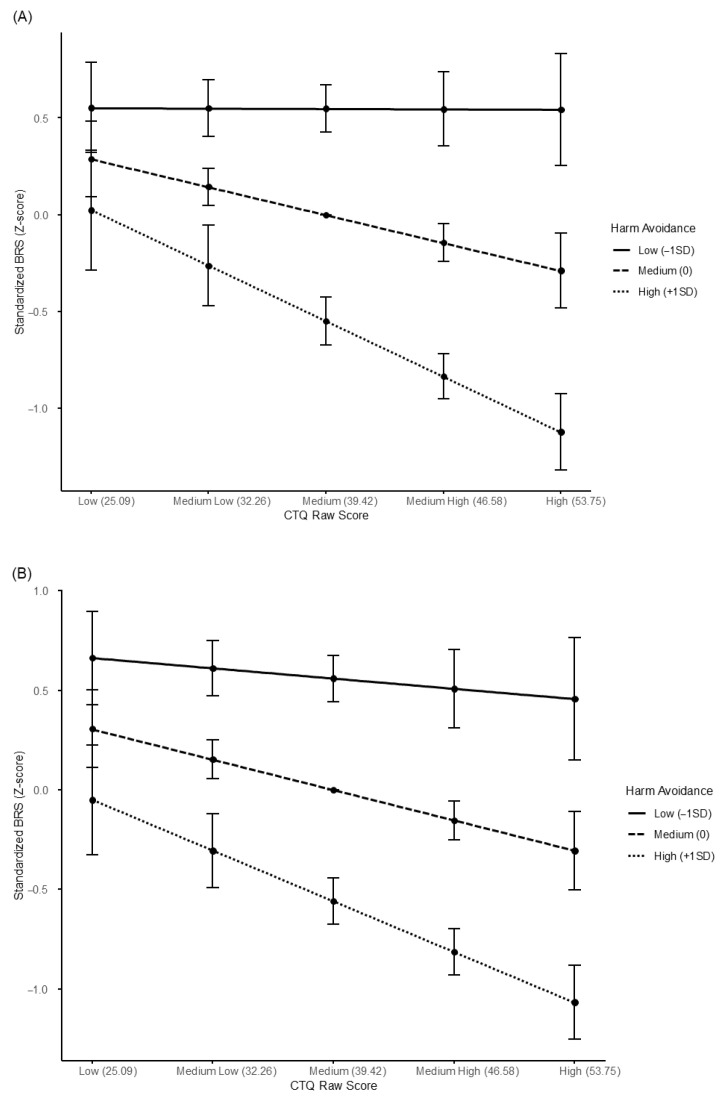
Moderating effect of harm avoidance in the (**A**) CTQ–BRS–PHQ-9 Model and (**B**) CTQ–BRS–GAD-7 Model.

As illustrated in Figure 2A,B, the strength of the indirect effects of CTQ on depression and anxiety through resilience varied according to HA levels. For depression (PHQ-9), the indirect effect was nonsignificant at low HA (−1 SD; β = −0.012, 95% CI [−0.125, 0.129]) but significant at mean (β = 0.122, 95% CI [0.015, 0.226]) and high HA (+1 SD; β = 0.256, 95% CI [0.111, 0.367]). For anxiety (GAD-7), the indirect effect was likewise nonsignificant at low HA (−1 SD; β = 0.042, 95% CI [−0.106, 0.214]) but significant at mean (β = 0.160, 95% CI [0.029, 0.289]) and high HA (+1 SD; β = 0.277, 95% CI [0.122, 0.407]). These findings indicate that the mediating effect of resilience was amplified among individuals with higher HA.

## 4. Discussion

To our knowledge, this study is among the earliest to test an integrated neuropsychiatric framework linking childhood trauma, resilience, and temperament in predicting adult depression and anxiety. The key finding was that childhood trauma was associated with lower resilience, and this indirect pathway was moderated by HA. Individuals with low HA appeared partially protected from this trajectory, whereas those with high HA exhibited amplified vulnerability. These findings support the proposed moderated mediation model and underscore the critical interplay between constitutional vulnerability (i.e., a stable temperament trait such as HA) and adaptive resources (resilience) in shaping trauma-related psychopathology.

Resilience (BRS) functioned as an indirect-only mediator in the relationship between childhood trauma (CTQ) and symptoms of depression (PHQ-9) and anxiety (GAD-7), with indirect effects observed at β = 0.122 (*p* = 0.025) and β = 0.160 (*p* = 0.017), respectively. Notably, in the anxiety model, although the total effect was nonsignificant, the indirect effect remained significant. This suggests that childhood trauma operates indirectly by weakening resilience rather than directly inducing symptoms. These findings align with prior research indicating that childhood trauma impairs resilience [15,24,25] and contributes to depression, anxiety, and suicidality [42]. Meta-analytic evidence further supports that resilience is inversely correlated with negative mental health outcomes, particularly under adverse conditions [43]. In addition, developmental research has long highlighted that resilience functions dynamically across the lifespan, shaped by cognitive, social, and environmental processes [24,25,26]. Extending this literature, the present study demonstrated that resilience may function not only as a stress buffer [18,44,45], but also as a dynamic mediating mechanism—akin to a “psychological immune system”—that explains how trauma translates into psychopathology [46,47]. These findings suggest that resilience should be prioritized as a key target in the assessment and treatment of individuals with a history of childhood trauma.

In this study, HA was selected as the primary TCI temperament variable due to its strongest evidence for links to reduced resilience and psychopathology. Consequently, this study demonstrated that HA strengthens the indirect pathway from childhood trauma to depression and anxiety by reducing resilience, with the effect being most pronounced among individuals with high HA (PHQ-9: β = 0.256, 95% CI [0.111, 0.367]; GAD-7: β = 0.277, 95% CI [0.122, 0.407]). Although mean symptom levels were modest, the PHQ-9 and GAD-7 distributions showed adequate variability to detect the observed indirect and moderated effects. Childhood trauma is consistently linked to higher risk-avoidance tendencies [22,23] and greater HA is associated with diminished resilience [21,22,23,27,28]. A large Finnish cohort study further reported that individuals with high risk-avoidance tendencies had a markedly increased risk of developing depression [31], and these traits were also associated with antidepressant discontinuation and poorer treatment responsiveness [48]. Although most prior studies have focused on associations, this study suggests that risk-avoidance temperament may moderate the pathway by which childhood trauma undermines resilience and contributes to psychopathology. This study advances prior resilience-focused frameworks by integrating constitutional and psychosocial mechanisms. This finding empirically supports conceptualizing risk avoidance as a temperamental vulnerability factor within the diathesis–stress framework. The finding that HA moderated the trauma–resilience pathway even within a relatively healthy adult sample suggests that this vulnerability mechanism may operate early, prior to the emergence of overt psychopathology. Clinically, these findings highlight the importance of assessing HA in trauma-exposed individuals, who may face heightened risk for reduced resilience, greater psychopathology, and poorer treatment response, with those exhibiting high HA representing a priority group for targeted, temperament-informed early interventions that can be incorporated into trauma–resilience models to guide more precise and effective neuropsychiatric care.

The study observation that HA moderates the impact of childhood trauma on resilience is novel but consistent with existing neurobiological and cognitive evidence. Prior studies have shown that both childhood trauma and high HA are associated with amygdala hyperresponsiveness and dysregulation within the insula–prefrontal circuitry, consequently impairing emotional regulation and stress recovery [49,50]. Such findings suggest overlapping neural substrates that may highlight both heightened threat sensitivity and diminished adaptive flexibility. These neural alterations align with behavioral patterns such as experiential avoidance and threat-oriented cognitive biases, which undermine resilience [6,39]. Conceptually, this supports frameworks that view resilience as a “psychological immune system” maintaining mental stability under adversity [46,47]. Therefore, the moderating role of HA observed in this study may reflect underlying cognitive and neural vulnerabilities through which trauma erodes adaptive capacity. Integrating the present findings with neurobiological evidence suggests that temperament-informed vulnerability models may clarify how trauma becomes biologically embedded [51]. Future studies incorporating behavioral measures alongside neuroendocrine and neuroimaging markers could help delineate the cognitive and neural mechanisms underlying this moderated mediation process.

In this context, resilience-enhancing approaches may be especially beneficial for individuals with childhood trauma—particularly those with high HA. First, therapies that reduce experiential avoidance and foster emotional openness, such as Acceptance and Commitment Therapy or self-compassion-based interventions, may counteract the maladaptive coping patterns associated with HA [52,53]. Second, interventions targeting cognitive biases—such as attentional control training or cognitive bias modification—could correct threat-focused information processing that compromises resilience [54,55]. Collectively, these implications position resilience as a modifiable treatment target and underscore the value of neuropsychiatrically informed, resilience-based interventions tailored to temperamental vulnerabilities. Future clinical trials are needed to determine whether these tailored interventions effectively reduce the long-term mental health risks associated with high HA. In parallel, resilience and emotional symptoms change over time, highlighting the importance of longitudinal and developmental designs to examine how temperament, resilience, and trauma-related symptoms co-evolve. Multi-wave or comparative studies, including clinical and non-clinical samples or groups with varying trauma exposure, would strengthen causal inference and help refine developmental neuropsychiatric models.

This study has some limitations. First, owing to the modest sample size and recruitment via public advertisements, the sample may not represent clinical populations or diverse sociodemographic backgrounds, thereby limiting generalizability. Second, the cross-sectional design precludes causal or temporal inferences among childhood trauma, resilience, and HA; therefore, longitudinal research is needed to examine developmental trajectories. Third, reliance on self-report measures (CTQ, BRS, PHQ-9, and GAD-7) introduces potential response bias. Although these instruments exhibit robust psychometric properties, future studies should incorporate clinician-rated assessments and neuroimaging or neuroendocrine biomarkers. Fourth, resilience is embedded within broader socio-environmental contexts—including social support, family functioning, and socioeconomic conditions [56,57,58]—yet these contextual determinants were not incorporated into our model. Their omission may narrow the interpretive breadth of our findings. Future research should include such factors to provide a more comprehensive account of resilience mechanisms. Finally, model fit indices indicated only modest fit (RMSEA > 0.10), thereby highlighting the need for replication and model refinement in larger, more heterogeneous samples.

## 5. Conclusions

This study suggests that resilience plays a central role in mediating the effects of childhood trauma on adult depressive and anxiety symptoms, with HA amplifying this indirect pathway. Individuals with high HA may represent a subgroup at heightened risk, thereby underscoring the need for tailored interventions that combine resilience enhancement with strategies targeting experiential avoidance and cognitive bias. Although the model fit was mixed and the samples were non-clinical, these findings provide an important foundation for future longitudinal and cross-cultural research aimed at refining trauma-informed and neuropsychiatrically grounded prevention and treatment approaches.

## Figures and Tables

**Table 1:** Participant characteristics.

Characteristic	Total
	(*N* = 218)
Age (years)	29.5 ± 6.02
**Sex**	
Male	79 (36.24)
Female	139 (63.76)
Level of education (years)	14.90 ± 1.73
**Occupational status**	
Employed	171 (78.44)
Unemployed	47 (21.56)
**Psychiatric Scale**	
CTQ	39.42 (14.33)
Emotional Abuse	7.59 (3.69)
Physical Abuse	8.04 (3.94)
Sexual Abuse	5.67 (2.16)
Emotional Neglect	10.60 (5.16)
Physical Neglect	7.51 (3.05)
**TCI**	
Novelty Seeking	35.46 (12.25)
Harm Avoidance	42.46 (15.63)
Reward Dependence	43.53 (11.02)
Persistence	41.27 (11.40)
BRS	19.82 (5.37)
PHQ-9	5.01 (5.48)
GAD-7	4.44 (4.52)

Data are presented as mean  ±  standard deviation or proportion (%). CTQ refers to the Childhood Trauma Questionnaire total score. Abbreviations: CTQ, Childhood Trauma Questionnaire; TCI, Temperament and Character Inventory; BRS, Brief Resilience Scale; PHQ-9, Patient Health Questionnaire-9; GAD-7, Generalized Anxiety Disorder-7.

**Table 2:** Correlation analysis of main variables.

Variables	CTQ	HA	BRS	PHQ-9	GAD-7
CTQ	1				
HA	0.381	1			
BRS	−0.421	−0.638	1		
PHQ-9	0.525	0.561	−0.596	1	
GAD-7	0.432	0.588	−0.573	0.736	1

Abbreviations: CTQ, Childhood Trauma Questionnaire; HA, Harm Avoidance; BRS, Brief Resilience Scale; PHQ-9, Patient Health Questionnaire-9; GAD-7, Generalized Anxiety Disorder-7.

**Table 3:** Mediation and moderated mediation effect of HA between CTQ and PHQ-9.

Path	β	SE	95% CI	*p*-Value
**Mediated model (Dependent variable: BRS)**				
CTQ → BRS	−0.147	0.057	[−0.255, −0.033]	0.011
HA → BRS	−0.561	0.055	[−0.657, −0.441]	<0.001
CTQ × HA → BRS	−0.161	0.041	[−0.222, −0.062]	<0.001
**Outcome model (Dependent variable: PHQ-9)**				
CTQ → PHQ-9	0.068	0.079	[−0.088, 0.222]	0.395
BRS → PHQ-9	−0.830	0.120	[−1.071, −0.600]	<0.001
**Effect decomposition (CTQ** **→** **PHQ-9)**				
Total effect	0.190	0.062	[0.067, 0.308]	0.002
Direct effect	0.068	0.079	[−0.088, 0.222]	0.395
Indirect effect (via BRS)	0.122	0.054	[0.015, 0.226]	0.025

Abbreviations: CTQ, Childhood Trauma Questionnaire; HA, Harm Avoidance; BRS, Brief Resilience Scale; PHQ-9, Patient Health Questionnaire-9; SE, standard error; CI, confidence interval.

**Table 4:** Mediation and moderated mediation effect of HA between CTQ and GAD-7.

Path	β	SE	95% CI	*p*-Value
**Mediated model (Dependent variable: BRS)**				
CTQ → BRS	−0.156	0.057	[−0.264, −0.042]	0.007
HA → BRS	−0.570	0.054	[−0.666, −0.453]	<0.001
CTQ × HA → BRS	−0.115	0.038	[−0.177, −0.026]	0.008
**Outcome model (Dependent variable: GAD-7)**				
CTQ → GAD-7	−0.091	0.081	[−0.249, 0.067]	0.260
BRS → GAD-7	−1.027	0.122	[−1.281, −0.801]	<0.001
**Effect decomposition (CTQ** **→ GAD-7)**				
Total effect	0.069	0.060	[−0.049, 0.186]	0.255
Direct effect	−0.091	0.081	[−0.249, 0.067]	0.260
Indirect effect (via BRS)	0.160	0.066	[0.029, 0.289]	0.017

Abbreviations: CTQ, Childhood Trauma Questionnaire; HA, Harm Avoidance; BRS, Brief Resilience Scale; GAD-7, Generalized Anxiety Disorder-7; SE, standard error; CI, confidence interval.

## Data Availability

The data presented in this study are available upon request from the corresponding author. The data are not publicly available due to ethical restrictions.

## Supplementary Materials

The following supporting information can be downloaded at https://www.mdpi.com/article/10.3390/brainsci15121308/s1, Table S1: Korean Validation Study Normative Data for all measures.

## Abbreviations

The following abbreviations are used in this manuscript:

BRS	Brief Resilience Scale
BRS-K	Brief Resilience Scale—Korean version
CFI	Comparative Fit Index
CI	Confidence Interval
CTQ	Childhood Trauma Questionnaire
GAD-7	Generalized Anxiety Disorder-7
HA	Harm Avoidance
PHQ-9	Patient Health Questionnaire-9
RMSEA	Root Mean Square Error of Approximation
SD	Standard Deviation
SEM	Structural Equation Modeling
SRMR	Standardized Root Mean Square Residual
TLI	Tucker–Lewis Index
β	Standardized Regression Coefficient
