# Supplementary material for: Resilience as a Mediator Between Childhood Trauma and Adult Psychopathology: The Moderating Role of Harm Avoidance in Korean Adults
Source: Brain Sci. 2025 Dec 4;15(12):1308. doi: 10.3390/brainsci15121308 (PMC12731143; doi:10.3390/brainsci15121308)
Supplement: Supplementary file 1 [file brainsci-15-01308-s001.zip › brainsci-3996786-supplementary.pdf]

Supplementary Table S1. Korean Validation Study Normative Data for all measures.

Measure	Subscale	Number of Items	Possible Range	Korean Validation Mean (SD)	Cronbach's α	Reference
The Korean version of the Child Trauma Questionnaire-Short Form	Emotional Abuse	8	8-40	8.09(3.08)	0.89	Yu, J., et al, 2009 (N = 393)
	Physical Abuse	5	5-25	8.85(3.93)	0.82	
	Sexual Abuse	5	5-25	5.66(1.70)	0.79	
	Emotional Neglect	5	5-25	11.16(3.87)	0.80	
	Physical Neglect	5	5-25	7.93(2.37)	0.51	
The Korean version of the Temperament and Character Inventory-Revised Short	Novelty Seeking	20	0-80	27.66(9.63)	0.84	Maumsarang Manual, 2007 (N = 2021)
	Harm Avoidance	21	0-84	35.18(10.35)	0.84	
	Reward Dependence	20	0-80	42.51(8.37)	0.77	
	Persistence	20	0-80	43.55(10.04)	0.85	
The Korean version of the Brief Resilience Scale		6	6–30	18.05(3.28)	0.94	Kim, J., et al. 2023 (N = 14,522)
The Korean version of the Patient Health Questionnaire-9		9	0-27	2.36(3.85)	0.88	Kim, M., et al, 2023 (N = 6022)
The Korean adaptation of the Generalized Anxiety Disorder-7		7	0-21	2.34(2.96) *	0.93	Ahn, J.K. et al., 2019 (N = 684) *

*Values refer to the subgroup without any mental disorder
